# Supplementary material for: Rapid Change in FcεRI Occupancy on Basophils After Venom Immunotherapy Induction
Source: Int J Mol Sci. 2025 Aug 4;26(15):7511. doi: 10.3390/ijms26157511 (PMC12346946; doi:10.3390/ijms26157511)
Supplement: Supplementary file 1 [file ijms-26-07511-s001.zip › ijms-3769482-supplementary.pdf]

Supplemental Materials -

Journal: International Journal of Molecular Science

Rapid change in FcεRI occupancy on basophils after ultra-rush venom immunotherapy induction

Viktoria Puxkandl[1,2], Stefan Aigner[1,2], Teresa Burner[1,2], Angelika Lackner[1,2], Sherezade Monino Romero[3,4], Susanne Kimeswenger[1,2], Wolfram Hoetzenecker[1,2], Sabine Altrichter[1,2,3,4]

1) [1] Department for Dermatology and Venerology, Kepler University Hospital, 4020 Linz, Austria; viktoria.puxkandl@kepleruniklinikum.at (V.P.); stefan.aigner@kepleruniklinikum.at (S.A.); teresa.burner@jku.at (T.B.); angelika.lackner_1@jku.at (A.L.); susanne.kimeswenger@jku.at (S.K.); sabine.altrichter@kepleruniklinikum.at (S.A.)
2) [2] Center for Medical Research (ZMF), Johannes Kepler University, 4020 Linz, Austria
3) [3] Institute of Allergology, Charité-Universitätsmedizin Berlin, Corporate Member of Freie Universität Berlin, Humboldt-Universität zu Berlin, 10117 Berlin, Germany; sherezade.monino-romero@charite.de
4) [4] Fraunhofer Institute for Translational Medicine and Pharmacology (ITMP), Immunology and Allergology (IA), 12203 Berlin, Germany
5) [5] Clinical Research Institute for Inflammation Medicine, Medical Faculty, Johannes Kepler University, 4020 Linz, Austria

Repository Tables

	Dose (µg)	Time (h)
**Day 1**	0.02	0
	0.04	1
	0.08	2
	0.2	3
**Day 2**	0.4	0
	0.8	1
	2	2
	4	3
**Day 3**	6	0
	8	1
	10	2
**Day 4**	8[#]	0
	10	1
	20	2
	40	3
	60	4
	80	5
	100	6

Table S1. Rush VIT induction dosing scheme.
[#]Indicates the individual dose adaption in the displayed patient due to systemic adverse reaction.

	Wasp n=13			Bee n=6		
	Before VIT	After VIT Induction (Ultra-Rush)	p-value	Before VIT	After VIT Induction (Ultra-Rush)	p-value
**Tryptase (µg/l)**	5.7 (4.9)	5.9 (4.6)	.10	4.62 ±2.19	4.43 ±2.38	.42
**Total FcεRI**	188783.89 ±126273.33	183701.34 ±126377.67	.35	273150.4 ±109367.23	26122.03 ±134118.04	.37
**Unoccupied FcεRI**	5163.45 ±5007.5	5642.86 ±4538.85	.21	5786.37 ±3909.37	6862.98 ±3427.61	.10
**Ratio unoccupied/total FcεRI[a]**	.072 ± .098	.081 ±.105	.01**	.021 (.02)	.026 (.031)	.08
**Soluble FcεRI (ng/ml)**	.58 (1.03)	.57 (1.98)	.01**	0.53 ±0.15	0.47 ±0.14	.10

Table S2 - Values before and after VIT. Statistical analysis was performed with a paired t-test or Wilcoxon-test (if no normally distributed). Normally distributed values are depicted as median ± standard deviation; not normally distributed values are displayed as median (interquartile range). [a]Since 2 patients did not have a detectable level of unoccupied FcεRI, the ratio could not be calculated.
Abbreviations: VIT – venom immunotherapy, SD – standard deviation, IQR – interquartile range.

	**Wasp (n=10)**		**Bee (n=5)**		**All (n=15)**	
	% Change EC_{50}	p-value	% Change EC_{50}	p-value	% Change EC_{50}	p-value
**Δ Total FcεRI**	.576	.08	**.900**	.04*	**.764**	<.01**
**Δ Unoccupied FcεRI**	.432	.21	-.700	.19	-.070	.81
**Δ ratio unoccupied/total FcεRI**	.333[a]	.42	**-.900**	.04*	-.181[a]	.55

Table S3. Changes in BAT sensitivity correlated with FcεRI changes before/after VIT induction.
Statistical analysis was performed with a Spearman-Rho. Shown values are correlation coefficient and p-value.
[a]Since 2 patients did not have a detectable level of unoccupied FcεRI, the ratio could not be calculated.
Abbreviation: EC_{50} - Half-maximum basophil activation

Repository Figures

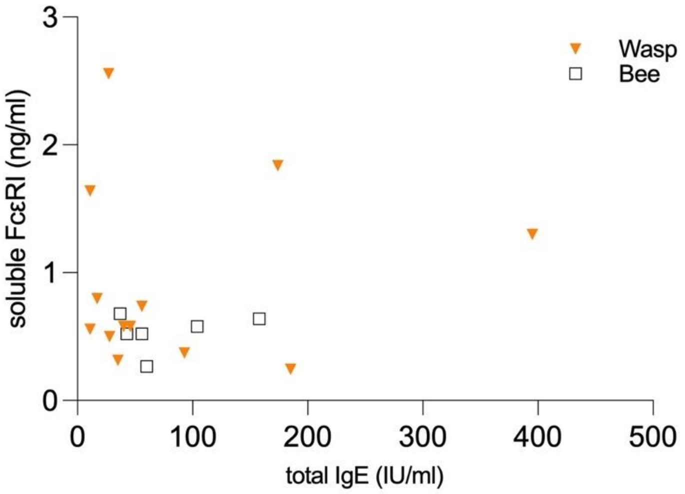

Figure S1. Correlation of sFcεRI and total IgE. When performing statistical analysis with Spearman-Rho - a correlation coefficient of -.144 (p-value .56) is seen.

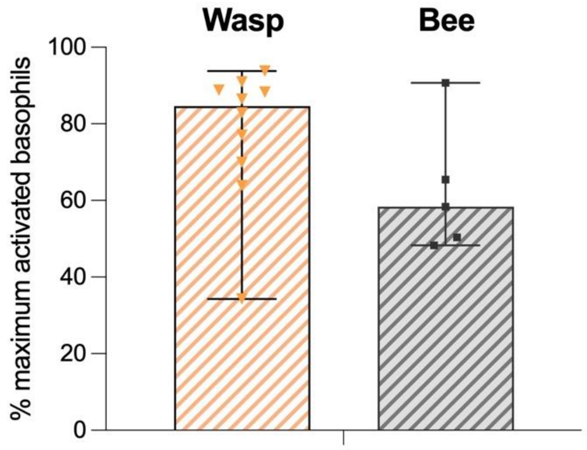

Figure S2. Maximum activated basophils (during BAT) before VIT induction.
Group comparison with unpaired t-test (normally distributed values) – p=0.07

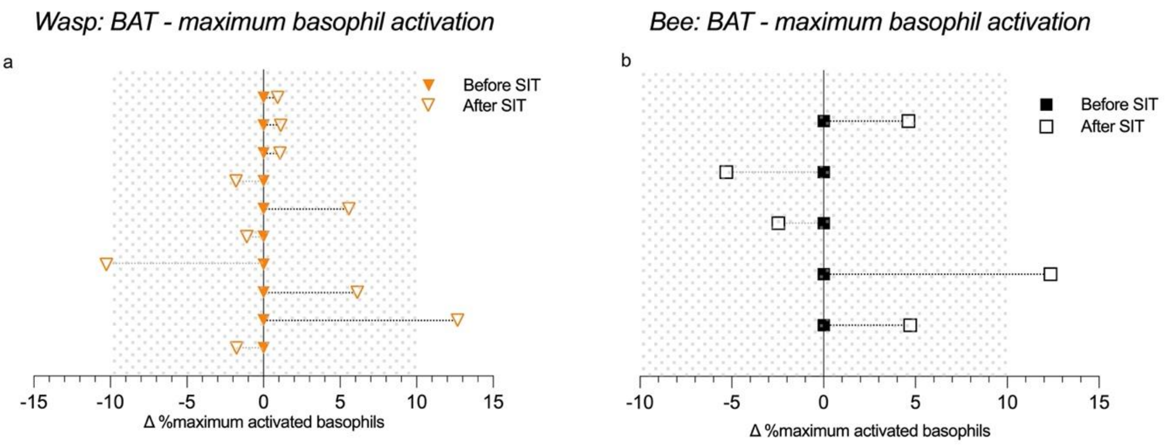

Figure S3 - Change of percentage of maximum activated basophils during BAT before and after VIT.

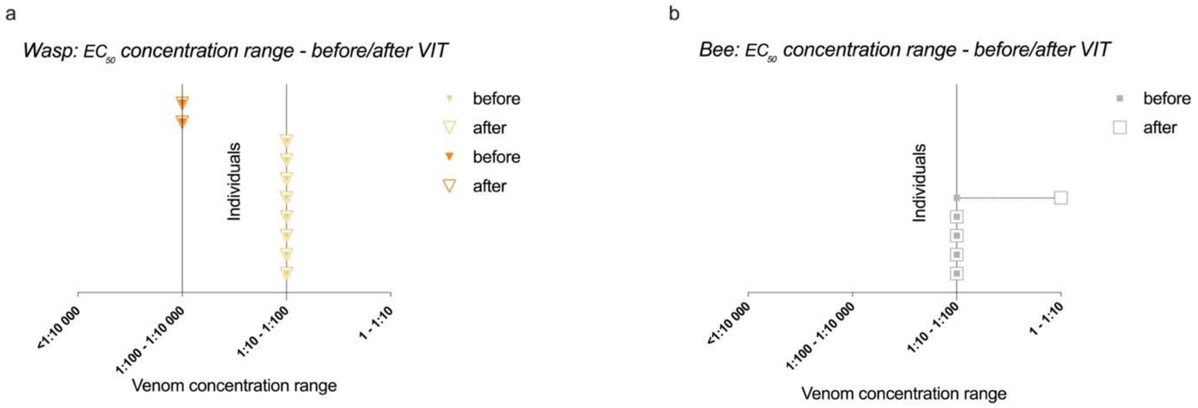

Figure S4. Change of EC_{50} dilution range after VIT induction.
The concentration equals 100µg/ml of the respective venom.
a) Change of concentration range of half-maximum activation (EC_{50}) in the basophil activation test (BAT) in wasp sensitized patients before venom immunotherapy (VIT)
b) Change of concentration range of half-maximum activation (EC_{50}) in the basophil activation test (BAT) in bee sensitized patients before venom immunotherapy (VIT)

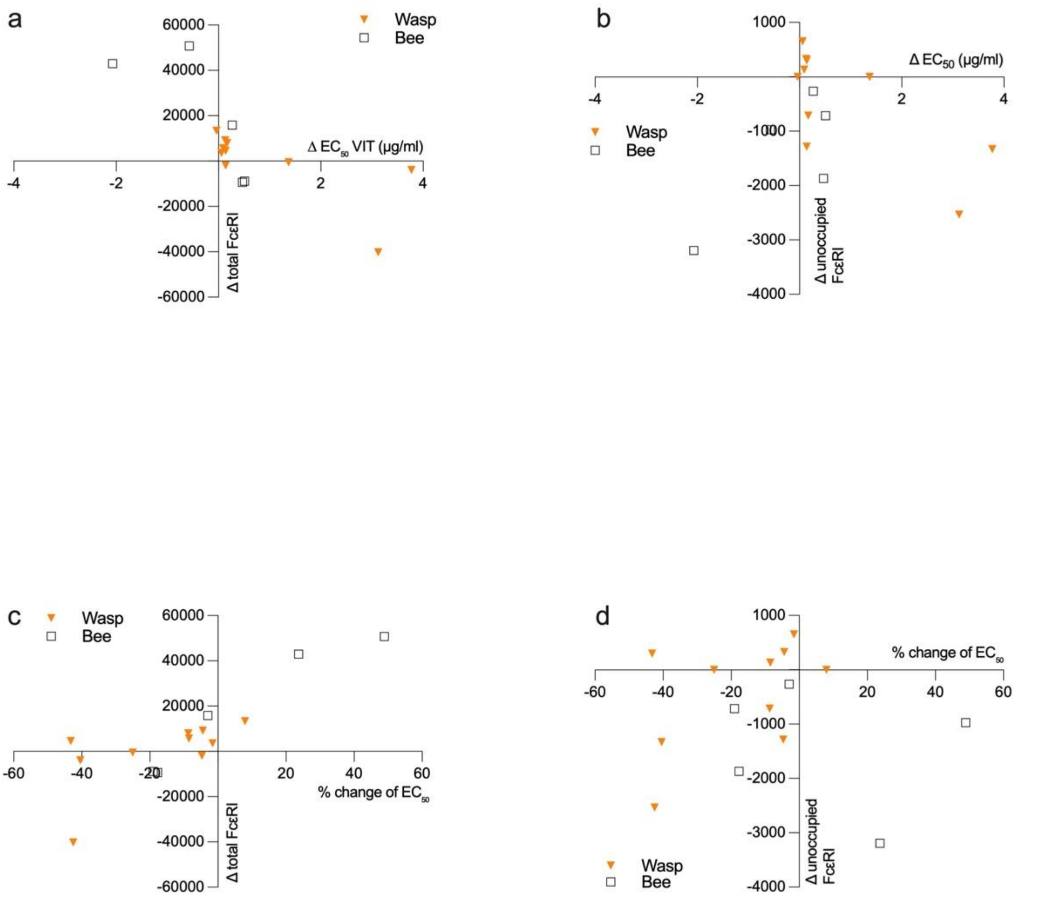

Figure S5. Correlation of absolute and relative EC_{50} change and FcεRI
Correlation of delta of EC_{50} (before/after VIT induction) with total and unoccupied and soluble FcεRI (a,b);
Correlation of relative change of EC_{50} (before/after VIT induction) with total and unoccupied (c,d)

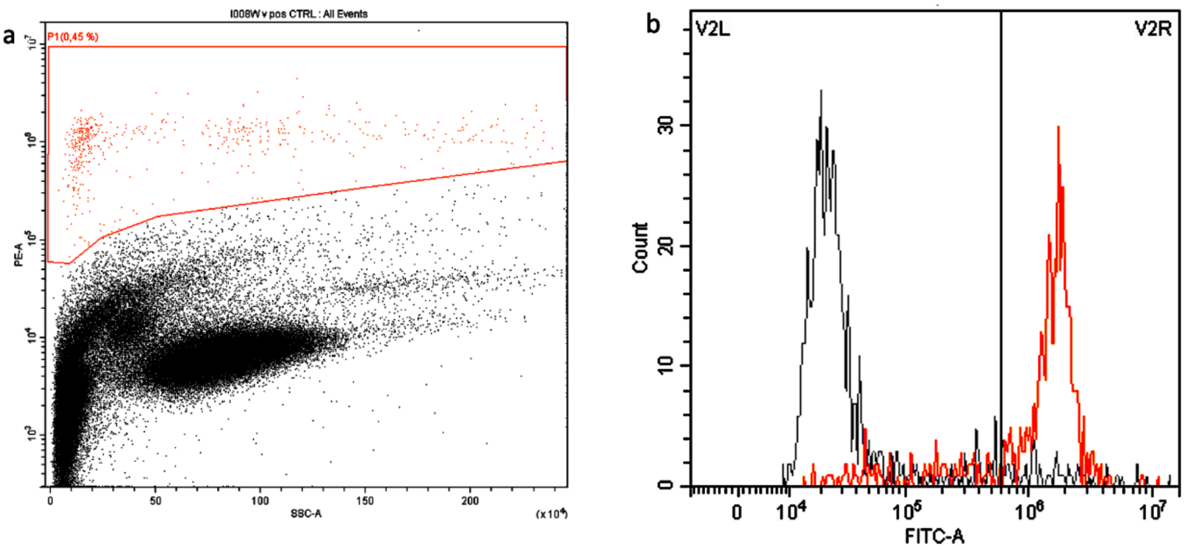

Figure S6a. *Gating strategy basophil activation test.*
A standardized basophil activation test kit from EXIBO© (BasoFlowEx Kit Ref. ED7043) was used (bee and wasp allergen from the company ALK (ALK wässerig SQ© 801 Bienengift 100µg/ml & ALK wässerig SQ© 802 Wespengift 100µg/ml)). After stimulation according to the kit the probes were analyzed by flow cytometry (Beckman coulter DxFlex©).
For analyzing the FACS data, the application CytExpert© was chosen. The used Gain settings are stated here: FSC, SSC, CD63 (FITC) , CD203c (PE). The Compensation Matrix was not altered (= 0,00 for all values of the Compensation Matrix).

b) The read out of the basophil activation test is the percentage of CD63 positive cells (red curve). The gating was set at the 5th percentile of the negative control (black curve), below which 95% of the unstimulated basophils fall.

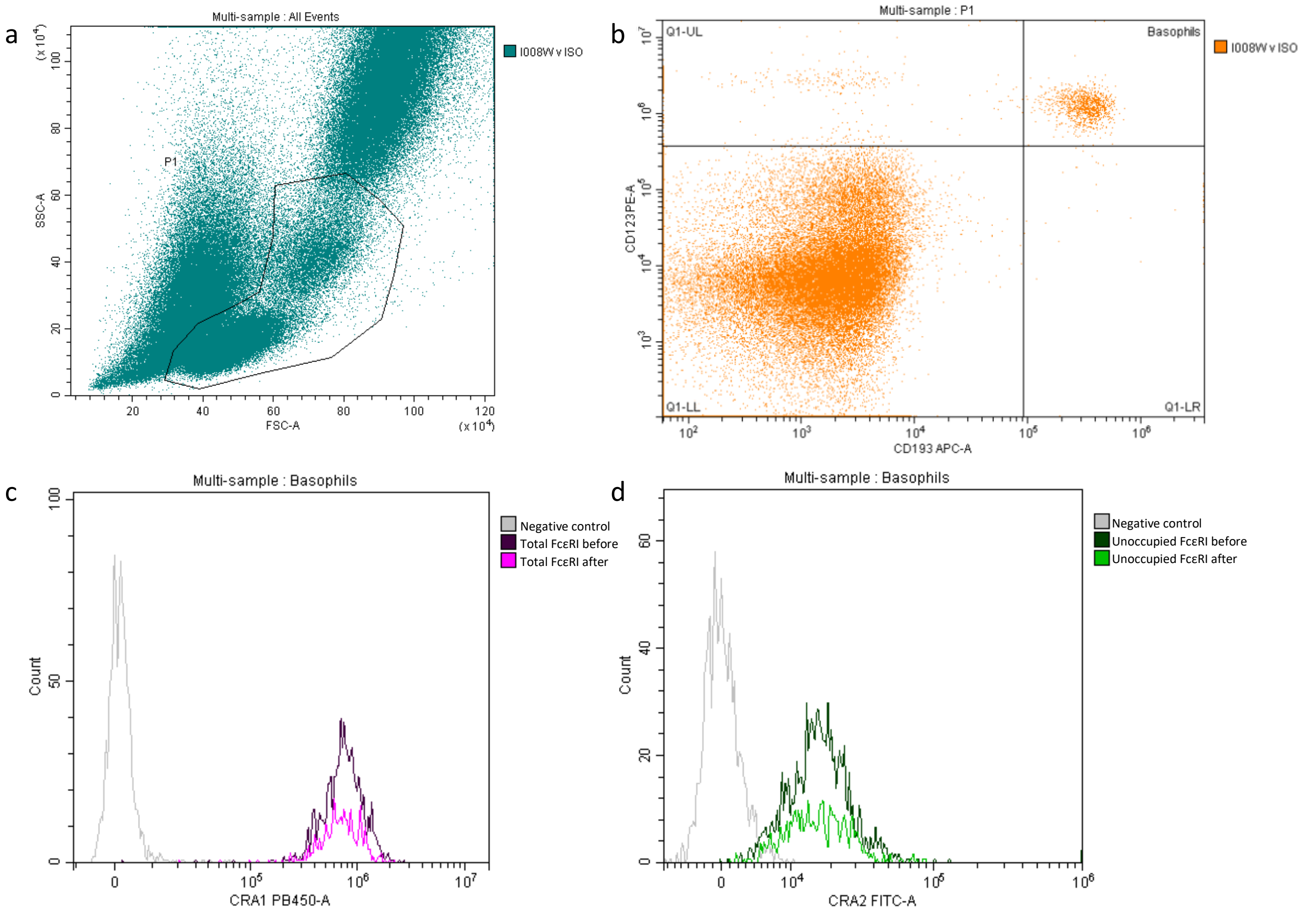

Figure S6b. *Gating strategy total and unoccupied FcεRI (before and after VIT induction)*

As a blocking solution, we used Human immunoglobulin - IG VENA 50g/l (Kedrion Bolognana, Italy). For our FACS protocol, anti-human CD193-APC (5E8, BD. Ref. 558208) and anti-human CD123-PE (9F5, BD. Ref. 555644) for the APC and PE Channels were used. The FcεRI were stained with CRA1-BV421 (334624, BioLegend®; staining of total FcεRI) and CRA2-FITC (GTX00853, GeneTex©; staining of unoccupied FcεRI) antibodies. The CRA1 antibody stains all FcεRI whereas the CRA2 antibody stains the IgE unoccupied FcεRI. Isotype control antibodies were IgG2b-BV421 (MPC-11, Biolegend. Ref. 400307) and IgG1-FITC (R&D Systems, IC002F).

CytExpert© was used for analyzing the FACS data. The used Gain settings are stated here: FSC, SSC, CD63 (FITC), CD203c (PE), APC, PB450. The compensation matrix was not altered (values = 0,00) except for FITC-PE (1,00) and PB450-APC (1,50).

a) With FSC on the X-axis and SSC on the Y-axis, the basophil containing leukocytes were selected.

b) These selected leukocytes were then further divided by sorting them according to their CD193-APC and CD123-PE signals, marking the basophil population.

c,d) The median flourescence intensity (MFI) was calculated for CRA1 (total FceRI, magenta curve (dark before; light after)) and CRA2 (free FceRI, green curve (dark before; light after)) individually in each patient. In the histogram above, the negative control is visualized with the gray curve.
